# Atrial fibrillation in a patient with Zika virus infection

**DOI:** 10.1186/s12985-018-0938-2

**Published:** 2018-01-25

**Authors:** Ligia Fernandes Abdalla, João Hugo Abdalla Santos, Renata Teodora Jales Barreto, Erick Martins e Souza, Fabrício Fonseca D’Assunção, Márcio Aurélio Borges, Valdinete Alves Nascimento, George Allan Villarouco da Silva, Victor Costa de Souza, Rajendranath Ramasawmy, Ana Carolina Campi-Azevedo, Jordana Graziela Coelho-dos-Reis, Lis Ribeiro do Vale Antonelli, Andréa Teixeira-Carvalho, Olindo Assis Martins-Filho, Felipe Gomes Naveca

**Affiliations:** 1 Programa de Pós-Graduação em Imunologia Básica e Aplicada, Universidade Federal do Amazonas, Manaus, Amazonas Brazil; 2 Universidade do Estado do Amazonas, Manaus, Amazonas Brazil; 3 Universidade Federal do Amazonas, Manaus, Amazonas Brazil; 4 Hospital Adventista de Manaus, Manaus, Amazonas Brazil; 5 Laboratório de Ecologia de Doenças Transmissíveis na Amazônia, Instituto Leônidas e Maria Diane – Fiocruz Amazônia, Manaus, Amazonas Brazil; 6 Programa de Pós-Graduação em Biologia Celular e Molecular, Instituto Oswaldo Cruz, Fiocruz, Rio de Janeiro, Brazil; 7 Fundação de Medicina Tropical – Dr Heitor Vieira Dourado, Manaus, Amazonas Brazil; 8 Universidade Nilton Lins, Manaus, Amazonas Brazil; 9 Instituto René Rachou – Fiocruz Minas, Minas Gerais, Brazil; 10 Programa de Pós-Graduação em Biologia da Interação Patógeno-Hospedeiro, Instituto Leônidas e Maria Diane – Fiocruz Amazônia, Manaus, Amazonas Brazil

**Keywords:** Zika virus, Arboviruses, Atrial fibrillation, Cardiac disorders

## Abstract

**Background:**

Zika virus is an emerging arbovirus of the family *Flaviviridae* and genus *Flavivirus* that until 2007 was restricted to a few cases of mild illness in Africa and Asia.

**Case presentation:**

We report a case of atrial fibrillation disclosed during an acute Zika virus infection in a 49-year-old man. Different biological samples were analyzed for the molecular diagnosis of Zika by real-time PCR, however only the saliva specimen was positive. The patient’s wife tested positive in the serum sample, although she was an asymptomatic carrier. Moreover, a complete overview of patient’s biomarkers, including cytokines, chemokines, and growth-factors levels, was analyzed and compared to gender and age matching non-infected controls, as well as other Zika infected patients, considering the 95%CI of the mean values. Elevated levels of CXCL8, CCL11, CCL2, CXCL10, IL-1β, IL-6, TNF-α, IFN-γ, IL-17, IL-1Ra, IL-4, IL-9, FGF-basic, PDGF, G-CSF, and GM-CSF were observed in the Atrial fibrillation patient, in contrast to uninfected controls. Furthermore, increased levels of CCL5, IL-1β, TNF-α, IFN-γ, IL-9, G-CSF, and GM-CSF were observed only in the atrial fibrillation patient, when compared to other Zika patients.

**Conclusions:**

To our knowledge, this is the first description of this type of cardiac disorder in Zika patients which may be considered another atypical manifestation during Zika virus infection.

## Background

Zika disease is an emerging illness caused by an arbovirus of the family *Flaviviridae* and genus *Flavivirus* [1]. In 1947, Zika virus (ZIKV) was isolated from a rhesus monkey in the Zika forest in Uganda, and 5 years later, in 1952, it was described infecting humans [2, 3]. Until 2007, ZIKV was restricted to a few cases of mild illness in Africa and Asia, and then it was associated with an outbreak of acute febrile illness in the Yap Island, Micronesia [4]. In Brazil, the virus was identified in autochthonous cases in the first months of 2015 [5, 6]. In August of the same year, an increase in the number of neonates with microcephaly was detected in Brazil and the hypothesis that ZIKV infection caused the microcephaly epidemic was formulated [7]. In November 2015, the Ministry of Health declared the situation a national public health emergency and a few months after this, WHO elevates this alert as a Public Health Emergency of International Concern [8–10].

ZIKV infections are often associated with slight fever, headache, maculopapular rash, arthralgia and conjunctivitis [4]. However, atypical clinical manifestations have been previously reported including neurological complications [11], congenital syndrome [12], ocular problems [13] and cardiovascular complications [14]. Here, we report a case of atrial fibrillation during a confirmed case of acute ZIKV infection.

## Case presentation

JLLP, a 49-year-old man, industrial worker, resident in Manaus, Amazonas, Brazil, was admitted to the emergency at Hospital Adventista de Manaus (HAM). The patient showed skin rash, pruritus, arthralgia, headache, myalgia, bilateral conjunctivitis, fever (38.5 °C) and hypertensive crisis with blood pressure (BP) of 240/120 mmHg, but heart rate and cardiac auscultation were normal. The patient had no travel history and described the appearance of symptoms 3 days before seeking medical attention. Besides, the patient reported the absence of hypertensive episodes or any other cardiac disorder in the past.

Immediately, the treatment for the hypertensive crisis was initiated with sodium nitroprusside (250 ml glycated serum 5% + Nipride – 1 ampoule = 2 ml) administered 5 ml/h by continuous infusion. In the following two, three and 4 hours it was administered 7 ml, 10 ml and 15 ml of sodium nitroprusside, respectively, but the blood pressure was still elevated. No abnormalities in electrocardiogram (ECG) and chest radiography (CR) were observed.

The patient was still refractory to blood pressure control (BP 238/120 mmHg) 4 hours after starting treatment, and showed elevated blood glucose levels (250 mg/dL), therefore, he was transferred to the Intensive Care Unit (ICU). Suddenly, the patient suffered a cardiac arrhythmia (atrial fibrillation - AF) which was chemically reversed with an attack dose of two ampoules (6 ml) of intravenous amiodarone hydrochloride (50 mg/ml). For the maintenance dose, six ampoules of amiodarone (8 ml/h) were administered in 5% glycated serum (250 ml) by continuous infusion for 12 h.

Due to the symptoms presented at the time of attendance, and the ongoing Zika outbreak in course, the patient and his wife, an asymptomatic contact, were inserted into the protocol for ZIKV surveillance. Both had samples of blood, urine, and saliva collected for arboviral testing by the reverse transcription real-time polymerase chain reaction (RT-qPCR).

On the sixth day of hospitalization, the patient underwent magnetic resonance imaging (MRI); echocardiographic doppler (DE) and coronary angiography (CA). Only the MRI was altered with bilateral supratentorial microangiopathic gliosis. A second ECG was performed on the eighth day of hospitalization, which presented no alterations and the patient was discharged. Serological tests for other infectious diseases were negative and the RT-qPCR results showed positivity for ZIKV in the saliva sample. Although still asymptomatic, his wife also tested positive for ZIKV in the serum sample.

## Materials and methods

The patient’s blood sample was collected for Dengue virus NS1 testing (CTK Biotech OnSite rapid test) and IgM/IgG serologic testing using enzyme immunoassay technology for dengue (Serion Elisa classic Dengue). The sample was also tested for rubella (Abbott AxSYM); measles (Bio-Rad Laboratories, Hercules, CA) and Parvovirus B19 (Serion Elisa), as recommended by the manufacturers’.

The serum, urine and saliva samples of the patient and his wife were sent to Instituto Leônidas e Maria Deane - Fiocruz Amazônia - to test for ZIKV [15]; Chikungunya virus (CHIKV) [16]; DENV [17]. A multiplex protocol also evaluated serum samples for Mayaro virus (MAYV) and Oropouche virus (OROV) infection [18].

The patient’s serum was also sent to Instituto René Rachou (Fiocruz Minas Gerais) for the analysis of cytokines, chemokines, and growth-factors levels. High-performance microbeads 27-plex assay (Bio-Rad, Hercules, CA, USA) was employed for detection and quantification of multiple targets, including: CXCL8 (IL-8); CXCL10 (IP-10); CCL11 (Eotaxin); CCL3 (MIP-1α); CCL4 (MIP-1β); CCL2 (MCP-1); CCL-5 (RANTES); IL1- β, IL-6, TNF- α, IL-12; IFN- γ, IL-17; IL-1Ra (IL-1 receptor antagonist); IL-2; IL-4; IL-5; IL-7; IL-9; IL-10; IL-13; IL-15; FGF-basic; PDGF; VEGF; G-CSF and GM-CSF. The sample was tested according to the manufacturer’s instructions on a Bio-Plex 200 instrument (Bio-Rad). The patient’s results were compared to two reference groups: I) a control group consisting of 54 healthy male subjects, age ranging from 18 to 40 years (median = 30 years), all living in Manaus – AM and II) a group consisting of 24 ZIKV-infected male patients, with classical zika illness presentation, age ranging from 20 to 59 years (median = 37 years), all living in Manaus – AM. The mean values for each biomarker were compared with the 95%CI values of each reference groups.

Supplementary exams included: hematological and biochemical tests; electrocardiogram (ECG); chest radiography (CR); abdominal ultrasonography (AU); coronary angiography (CA); doppler echocardiographic (DE) and magnetic resonance imaging (MRI).

## Results

### Serology and molecular tests

The DENV NS1 testing was negative, as well as the serological tests for rubella, parvovirus B19, and measles IgM, whereas positive serological results were observed for rubella and measles IgG.

The RT-qPCR showed positivity to ZIKV in the patient’s saliva (mean Ct value: 32.3) and in the serum of his wife (mean Ct value: 31.1). No positivity was found for DENV, CHIKV, MAYV or OROV.

### Cytokine, chemokine and growth factors levels

**Fig. 1:**
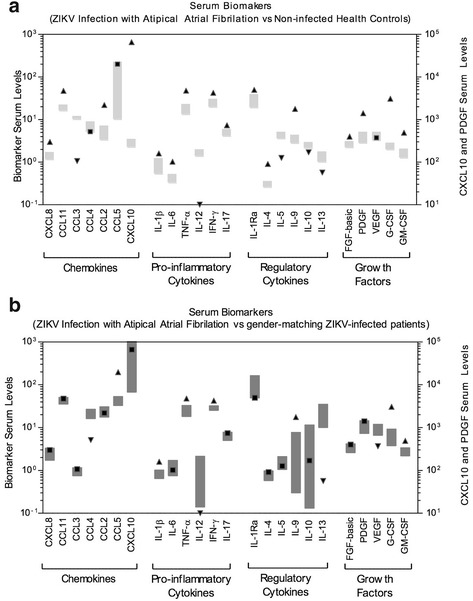
Overview of serum chemokines, cytokines and growth factors of the atrial fibrillation patient compared to control groups. Serum biomarkers: CXCL8 (IL-8); CXCL10 (IP-10); CCL11 (Eotaxin); CCL3 (MIP-1α); CCL4 (MIP-1β); CCL2 (MCP-1); CCL-5 (RANTES); IL1-β, IL-6, TNF-α, IL-12; IFN-γ, IL-17; IL-1Ra (IL-1 receptor antagonist); IL-2; IL-4; IL-5; IL-7; IL-9; IL-10; IL-13; IL-15; FGF-basic; PDGF; VEGF; G-CSF and GM-CSF were measured by high performance microbeads 27-plex assay, as described in materials and methods, with the 95%CI represented by gray boxes. Black arrows represent an increase (up) or decrease (down) in the patient’s biomarkers level in comparison to control groups; a black square represents biomarkers levels at the 95%CI. **a** Biomarkers’ levels: case vs. gender matching non-infected heath controls; (**b**) Biomarkers’ levels: case vs. gender matching ZIKV-infected patients

The serum levels of chemokines, cytokines and growth factor were evaluated and the data presented in Fig. 1. The results demonstrated that there was an increase in the levels of chemokines (CXCL8, CCL11, CCL2 and CXCL10); pro-inflammatory cytokines (IL-1β, IL-6, TNF-α, IFN-γ and IL-17); regulatory cytokines (IL-1Ra, IL-4 and IL-9) and growth factors (FGF-basic, PDGF, G-CSF and GM-CSF) in the ZIKV-infected patient with atrial fibrillation, considering the 95%CI of the mean values observed for a control group of gender-matching healthy individuals. No difference was observed for the CCL4 and CCL5 (chemokines) and VEGF (growth factor). Conversely, lower levels of CCL3, IL-12, IL-5, IL-10, IL-13 were observed in the ZIKV-infected patient with atrial fibrillation as compared to the healthy controls (Fig. 1a).

The overall biomarker profile observed in the ZIKV-infected patient with atrial fibrillation was also compared with a group of gender-matching ZIKV-infected patient. Data analysis revealed that the case reported here displayed increased the levels of CCL5, IL-1β, TNF-α, IFN-γ, IL-9, G-CSF and GM-CSF and decreased levels of CCL4, IL-12, IL-13 and VEGF as compared with the 95%CI of the mean values found in the group patients infected with the ZIKV. No differences were observed for the CXCL8, CCL11, CCL3, CCL2, CXCL10, IL-6, IL-17, IL-1Ra, IL-4, IL-5, IL-10, FGF-basic and PDGF (Fig. 1b).

### Supplementary exams

The blood samples for hematological and biochemical tests were collected during the acute phase of the disease, 4 days after the onset of symptoms, and showed: hematocrit: 46%; hemoglobin: 15 g/dl, leukocytes: 16,900 × 10^3^ mm^3^, platelets: 143,000 × 10^3^ mm^3^; lymphocytes: 26%; eosinophils: 2%; neutrophils: 88%; monocytes: 4%; ALT: 26 U/L; AST: 24 U/L; GGT: 32 U/L; creatinine: 0,78 mg/dL; urea: 25 mg/dL; alkaline phosphatase: 56 mg/dL; total bilirubin: 0,5 mg/dL; direct bilirubin: 0,2 mg/dL; indirect bilirubin: 0,3 mg/dL; creatine phosphokinase: 62 U/L; prothrombin time (PT):14,5 s; prothrombin activity: 84,5%; albumin: 3,2 g/dL and C protein: 21 mg/L.

The patient underwent two ECG, one performed during the hospitalization period and other 8 days after, without abnormalities. Among the image exams, only the MRI results showed an alteration, a bilateral supratentorial microangiopathic gliosis, which may be related to acute myocardial infarction.

## Discussion

In the present study, we described a case of AF in a patient with ZIKV infection, 4 days after the onset of symptoms. Until the present time, there is no similar reports relating cardiac arrhythmias and ZIKV virus infection. However, a case of myocarditis associated with to ZIKV infection during the acute phase of illness [14]. Considering that the patient of this study had no prior history of cardiovascular disease and no other heart abnormality was observed in the image exams, we may hypothesize the association of FA with the acute ZIKV infection.

Some studies have reported the relation of a cardiac disorder and arbovirus infection. In a survey carried out in a cardiovascular reference center during a dengue epidemic in Colombia (2010), 24 patients with dengue were related to cardiovascular symptoms, and 42.8% of these individuals had heart rhythm disturbances [19]. Another case report described West Nile virus causing myocarditis with an arrhythmia that led to patient death [20]. In 1972, a study described cases of Chikungunya related to cardiac manifestations such as myocarditis and cardiomyopathy [21].

Studies in rodents reinforce this theory, once changes in the ventricular repolarization were observed in ECG after ZIKV infection. Authors also found abnormalities in the synaptic conduction of motor neurons in the heart and cardiac muscles, and such changes could trigger arrhythmic processes [22]. Furthermore, myocarditis during ZIKV infections was also demonstrated in a mouse model [23], where multiple necrosis loci and myocarditis, possibly associated with pulmonary edema, were observed.

Different studies have reported the detection of viral RNA in various body fluids such as blood [24], saliva [25] and urine [26]. Indeed, Musso (2015) found that the ability to detect ZIKV RNA in saliva was higher compared to blood and recommend using different biological fluids to increase the sensitivity of the molecular detection of ZIKV. Based on these studies, and on our results where the ZIKV detection was higher in saliva samples (unpublished data), we chose to collect serum, urine and saliva samples. Interesting, only the patient’s saliva sample was positive in the RT-qPCR.

As some studies have pointed to asymptomatic cases of ZIKV infection through sexual contact [27] or blood transfusion [28], we decided to collect samples from the patient’s wife, an asymptomatic contact, and positivity was confirmed in the plasma sample. This result is fascinating since the literature is still very scarce regarding the asymptomatic cases of ZIKV infection.

Concerning the results found in hematological and biochemical tests, the patient presented mild thrombocytopenia (143,000 × 10^3^ mm^3^), leukocytosis (16,900 × 10^3^ mm^3^) and neutrophilia (88%). Comparing these findings with other flaviviruses, the frequent alterations observed in dengue infection are thrombocytopenia, hemoconcentration (with increased hematocrit) and leukopenia; leukocytosis is also described, but in elderly patients [29]. In Yellow fever, at the beginning of the disease, the blood count shows leukocytosis with neutrophilia and then evolves to leucopenia, lymphocytosis, and thrombocytopenia at later stages [30]. About 50% of the patients infected with West Nile virus present leukocytosis and other 15% shows leukopenia [31].

We observed alterations in the immune response with elevated levels of some cytokines, chemokines and growth factors in contrast to healthy controls. Other studies also found an increase of several pro-inflammatory cytokines including IL-18, TNF-α, IFN-γ, IL-8, IL-6, GRO-α and IL-17 [32, 33]. When compared to other individuals who are also in the acute phase of ZIKV infection, the patient also had elevated levels in different biomarkers. Other studies have observed a direct link between inflammation and atrial remodeling and, consequently, AF maintenance [34]. In general, the results revealed that the ZIKV-infected patient with atrial fibrillation presented a typical serum biomarker storm already reported for ZIKV-infected patients [33], with a more prominent pro-inflammatory status mediated by CCL5, IL-1β, TNF-α, and IFN-γ. This result links the inflammatory cytokines to the atrial fibrillation onset and prognostic implications, as previously reported [35]. Therefore, it is possible that the elevation of pro-inflammatory cytokines during ZIKV infection may increase the risk of AF.

## Conclusion

Our findings support the cardiac involvement as one of the atypical manifestations during ZIKV infections. The present results also strengthen the importance of collecting other body fluids in addition to serum, to improve the diagnostic of Zika. Besides, we describe several biomarkers levels altered in comparison to healthy controls and other Zika patients.

## Abbreviations

AF: Atrial fibrillation
BP: Blood pressure
CA: Coronary angiography
CHIKV: Chikungunya virus
CR: Chest radiography
DE: Echocardiographic doppler
DENV: Dengue virus
ECG: Electrocardiogram
HAM: Hospital Adventista de Manaus
ICU: Intensive Care Unit
MAYV: Mayaro virus
MRI: Magnetic resonance imaging
OROV: Oropouche virus
RT-qPCR: Reverse transcription real-time polymerase chain reaction
WHO: World Health Organization
ZIKV: Zika virus
